# Preeminent Flame-Retardant and Smoke Suppression Properties of PCaAl-LDHs Nanostructures on Bamboo Scrimber

**DOI:** 10.3390/molecules28114542

**Published:** 2023-06-03

**Authors:** Fei Yang, Ailian Hu, Chungui Du, Jiawei Zhu, Yuting Wang, Yuran Shao, Qichao Bao, Yin Ran

**Affiliations:** College of Chemistry and Materials Engineering, Zhejiang A & F University, Hangzhou 311300, China; yangfeier0826@163.com (F.Y.); hal15857832323@163.com (A.H.); wangyuting270229@icloud.com (Y.W.); shao18309819091@163.com (Y.S.); bqc1125573308@gmail.com (Q.B.); ry18119295807@163.com (Y.R.)

**Keywords:** bamboo scrimber, hydrotalcite, flame retardant, smoke suppression

## Abstract

Bamboo scrimber is widely used in interior decoration, architecture, and many other fields. However, it has caused huge security risks due to its inherent flammability and easy-to-produce toxic volatiles after combustion. In this work, the bamboo scrimber with superior flame retardant and smoke suppression properties was produced via the coupling of phosphocalcium-aluminum hydrotalcite (PCaAl-LDHs) with bamboo bundles. The results demonstrated that the flame-retardant bamboo scrimber (FRBS) heat release rate (HRR) and total heat release (THR) were, respectively, reduced by 34.46% and 15.86% compared with that of untreated bamboo scrimber. At the same time, the unique multi-layer structure of PCaAl-LDHs effectively slowed down the release rate of flue gas by extending its escape path. Cone calorimetry showed that the total smoke emissions (TSR) and specific extinction area (SEA) of FRBS were, respectively, reduced by 65.97% and 85.96% when the concentration of the flame retardant was 2%, which greatly developed the fire safety of the bamboo scrimber. This method not only improves the fire safety of bamboo scrimber but can also be expected to broaden its use scenarios.

## 1. Introduction

With the positive response of countries around the world to the initiative of becoming carbon neutral, biomass materials have ushered in a good opportunity for vigorous development. Due to the advantages of green environmental protection and sustainable development, the demand for biomass materials such as wood and bamboo has increased significantly in the fields of interior decoration and architecture. Among numerous wood and bamboo products, bamboo scrimber has become a current research hotspot due to its wide source of raw materials and excellent mechanical properties [1,2,3,4]. Owing to its intrinsic flammability, its increased use inevitably increases the risk of fire. For instance, in 2018, the National Museum of Brazil caught fire, and the wooden structural materials and flammable chemicals such as ethanol and methanol used to store specimens in the museum caused the flames to engulf almost all the rooms, resulting in the burning of 20 million items, causing serious economic losses. In 2019, the fire at Notre Dame in Paris destroyed the wooden roof and nearly destroyed hundreds of years of civilization. Therefore, improving the fireproof performance of bamboo scrimber has great practical significance for maintaining the safety of people’s lives and property and promoting the development of the industry.

As a bamboo-based fiber composite material, bamboo scrimber is composed of bamboo bundles as basic components and glued in a parallel arrangement [5]. However, bamboo scrimber is inflammable due to the raw nature of the material. Under high-temperature conditions, bamboo fibers and adhesives generate a large amount of heat and toxic gases, posing a threat to the safety of people’s lives and property [6]. In order to improve the fire resistance of bamboo scrimber, researchers have conducted a lot of research. Yu and coworkers [7] mixed glass fiber (GF) with bamboo fiber. The results showed that the limitation duration increased by 650% and the mass loss rate decreased by 400%, while the flame retardancy was greatly improved. Du and coworkers [8] explored the effect of the low-concentration ammonium polyphosphate impregnation (APP) sequence on the combustion properties of bamboo scrimber. These results showed that after being immersed in APP, the thermal stability of bamboo bundles was significantly increased. However, to expand the scope of the application of bamboo scrimber, it was not only necessary to improve its flame retardancy but also to reduce the amount of toxic smoke released. According to statistics, more than 80% of people are killed in fires due to the inhalation of toxic smoke or suffocation [9]. Therefore, it is urgent to develop flame retardants with both fire and smoke suppression properties.

Layered double hydroxides (LDHs) can provide a new way to solve these problems. LDHs are known as anionic clays and consist of positively charged metal layers and interlayer anions, as well as water [10]. Due to its large specific surface area and high dispersion of active sites, it has good adsorption performance, which makes it easier to bond with the substrate to form a stable structure [11,12]. Liu and coworkers [10] intercalated ammonium polyphosphate (APP) and acid red 88 (AR88) into LDHs. This greatly improved the flame retardancy and smoke suppression of PP. Abrishamkar and coworkers [13] prepared a modified multi-functional PU foam coating with LDHs, graphite oxide (GO), and graphite oxide modified by sulfonated sodium carbonate [4] arene (SC4A) as substrates. These results indicate that the PU had excellent flame retardancy and smoke suppression functions. Therefore, LDHs demonstrated great potential for application in the field of flame retardancy and smoke suppression.

In this work, to improve the fire properties and smoke suppression of bamboo scrimber, we synthesized layered calcium phosphate aluminum hydrotalcite (PCaAl-LDH) through the coprecipitation method and applied it to the flame retardant of the bamboo scrimber for the first time. PCaAl-LDH flame-retardant bamboo scrimber (referred to as FRBS) was prepared through the atmospheric pressure impregnation of PCaAl-LDH, glue impregnation, preforming, and hot pressing. The physical and mechanical properties, flame retardant, and smoke suppression properties of bamboo scrimber before and after treatment were investigated in detail. In short, this work aimed to investigate the effect of different concentrations of PCaAl-LDH on the flame retardancy and smoke suppression of bamboo scrimber, which could improve the fire safety performing bamboo scrimber effectively and facilitate the use of bamboo scrimber for construction and decorative materials.

## 2. Results and Discussion

### 2.1. Drug Loading Ratio of Flame-Retardant Bamboo Scrimber (FRBS)

**Figure 1:**
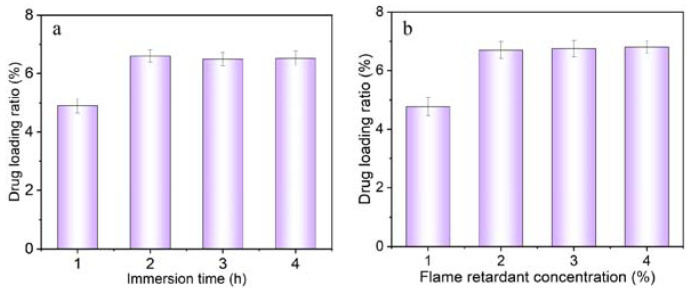
FRBS drug loading under various conditions. (**a**) Different immersion time, (**b**) Different flame retardant concentration.

This section adopted the control variable method to explore the influence of the atmospheric immersion process on the drug loading of bamboo bundles. This experimental design aimed to isolate and analyze the specific effects of the immersion process by controlling other variables. The relationship between the atmospheric immersion process and the drug-loading ratio of bamboo bundles is shown in Figure 1.

Figure 1a shows the drug loading rate of the bamboo bundle under different immersion times. With the increase in the immersion time, the drug loading of the bamboo bundle increased gradually and then tended to balance. Therefore, the inflection point of 2 h was determined as the best dipping time. As can be seen from Figure 1b, the loading rate of the bamboo bunches increased with the increase in the flame retardant fraction. According to the slope, when the flame-retardant concentration increased from 1% to 2%, the loading increased significantly, and when the flame-retardant concentration increased from 2% to 4%, the loading increased slowly. Therefore, combined with the production cost and benefit, 2% were selected for the follow-up flame-retardant bamboo scrimber experiment.

### 2.2. Microstructures of FRBS

**Figure 2:**
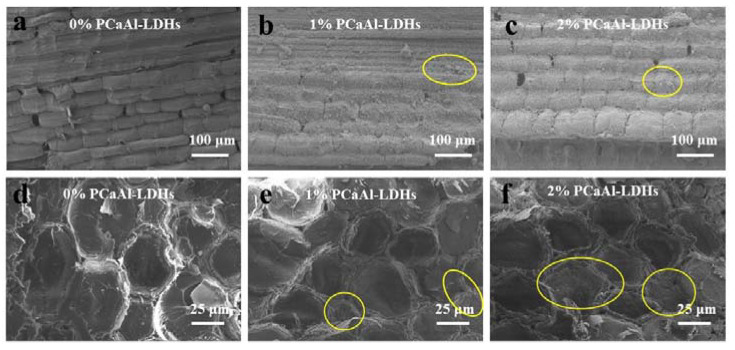
The SEM images of FRBS. (**a**–**c**) Longitudinal-section, (**d**–**f**) Cross-section.

To determine the impact of the flame retardant treatment on the microstructures of the bamboo bundles, SEM was used. Figure 2a–c displays the longitudinal-section SEM images of FRBS at varying concentrations of the flame retardant. These results indicate that after PCaAl-LDHs treatment, the vascular bundles and parenchyma cells retained their original morphology and microstructures, which was consistent with the conclusions drawn in the relevant literature [14,15]. A comparison of the scanning electron microscopy (SEM) images of longitudinal sections under different concentrations revealed a consistent and uniform coverage of powder. This observation signified the successful loading of the PCaAl-LDHs onto the parenchyma cells within the bamboo bundles. The cross-sectional resolve in Figure 2d–f. We can see from the yellow circle that cell walls and cavities were effectively covered with PCaAl -LDHs, which increased the filling or covering area corresponding to higher levels of flame-retardant concentration. Therefore, this observation indicated that PCaAlLDHs could easily enter the inside of the bamboo cells.

**Figure 3:**
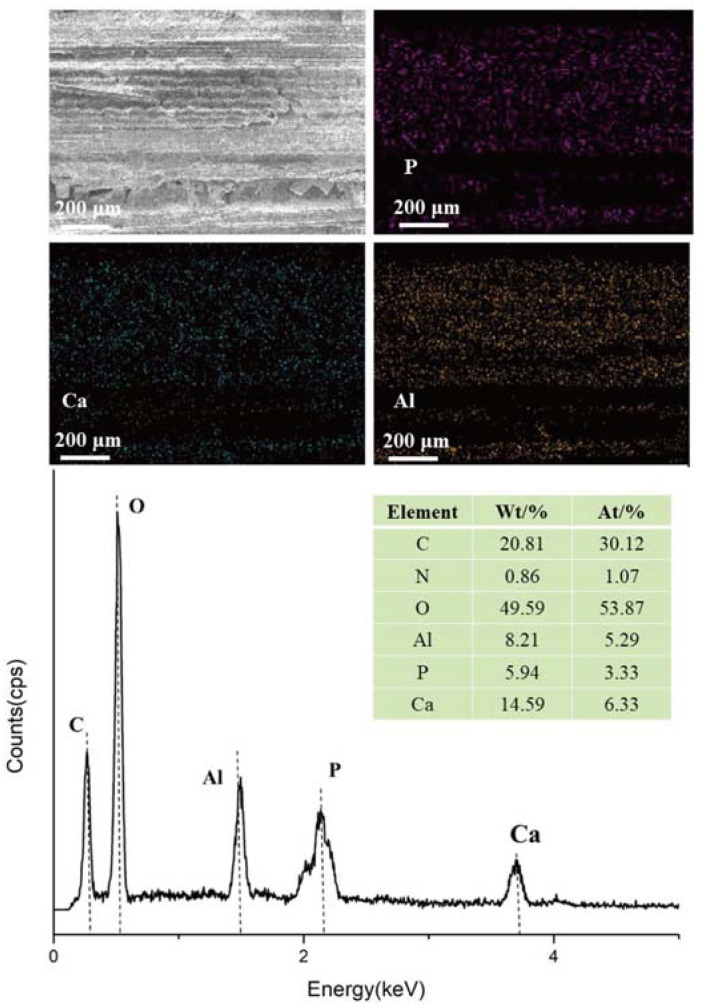
EDS spectra of FRBS.

Based on the above analysis, the concentration of the flame retardant was determined to be 2%, and the immersion time was 2 h. Additionally, 1% of the flame retardant concentration was selected as the control group to carry out the following experiment. The C, O, Al, P and Ca elements were observed from the EDS (Figure 3) of the surface of FRBS, with the element of C and O originating from the bamboo bundles [16]. EDS provided direct evidence that P, Ca, and Al were evenly distributed on the surface of the bamboo scrimber, which originated from PCaAl-LDHs. In short, the above analysis further confirmed that the PCaAlLDHs were located on bamboo bundles.

### 2.3. Physical and Mechanical Properties of FRBS

During the process of the flame-retardant treatment, it was crucial to take into account the potential influence of the flame retardant on the physical and mechanical properties of the bamboo scrimber. This consideration was of paramount importance in order to ensure the overall performance and functionality of the bamboo scrimber in various applications. Herein, FRBS was prepared using 1% and 2% PCaAl-LDHs flame retardants, and the original was prepared under the same hot pressing condition as the blank control. According to the standard “bamboo scrimber Floor” (GB/T 30364–2013), the density, water absorption expansion rate, and horizontal shear strength of FRBS were tested. Additionally, the untreated bamboo scrimber was taken as the counterpart.

**Figure 4:**
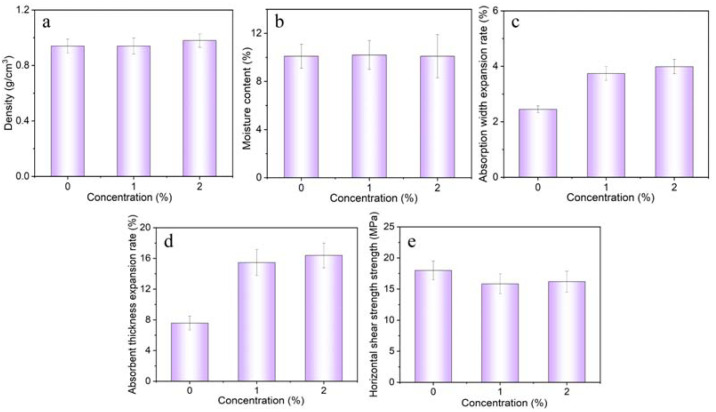
The mechanical and physical properties of FRBS. (**a**) Density, (**b**) Moisture content, (**c**) Absorption width expansion rate, (**d**) Absorbent thickness expansion rate, (**e**) Horizontal shear strength.

The impact of PCaAl-LDHs on the density and water content of the bamboo scrimber can be observed in Figure 4a,b. The results indicate that PCaAl-LDHs exhibited a negligible influence on both the density and the water content of bamboo scrimber. This suggested that the addition of PCaAl-LDHs did not significantly alter the physical characteristics related to the density and moisture absorption of the bamboo scrimber. On the other hand, Figure 4c,d illustrates the significant effects of flame retardants on the expansion rate of the absorbent width and thickness of bamboo scrimber. This is because the PCaAl-LDHs flame retardant primarily adhered to the surface of bamboo bundles through physical adsorption rather than forming chemical bonds with the cellulose fibers inside the bundles. The flame retardant itself had a relatively large specific surface area, which increased the water absorption and swelling capacity of the bamboo bundles [17]. These findings demonstrate that the inclusion of flame retardants had a substantial impact on the expansion rate of both the width and thickness of the bamboo scrimber material. Figure 4e shows that the flame retardant slightly affected the horizontal shear strength. The absorbent width expansion rate and absorbent width expansion rate of FRBS increased with the increase in the flame-retardant concentration. When the concentration of the flame retardant was 1% and 2%, the absorbent width of FRBS increased by 52.65% and 62.85%, respectively. Thereinto, the water absorption width expansion rate ≤4% and the horizontal shear strength ≤12% met the national standard. It was crucial to comprehensively assess and understand the influence of flame-retardant additives on the physical and mechanical properties of the bamboo scrimber. By gaining this understanding, informed decisions could be made regarding the suitability of flame-retardant additives for specific applications. This knowledge further enabled the development of flame-retardant treatments, which achieve a delicate equilibrium between meeting the fire safety requirements and preserving the desirable material characteristics. As a result, optimal performance, and reliability could be ensured when these materials were utilized in actual production and application.

In addition, the physical properties of bamboo scrimber treated when with water-based inorganic flame retardants prepared by other scholars were compared with our FRBS [18]. The results are shown in Table 1. Sample numbers 1 and 2 correspond to other water-based inorganic flame retardants treated bamboo scrimber and FRBS, respectively [18]. We selected two groups of data with similar drug loads for comparison. As can be seen from Table 1, when the drug loading of the water-based inorganic flame retardant was 3.98%, compared with the original material, the water content of the treated material increased by 39.5% and the swelling rate of the water absorption thickness increased by 300%. When the drug loading of PCaAl-LDHs was 4.81%, the water content of the treated material increased by 0.9% and the swelling rate of the water absorption thickness increased by 103.95%. Obviously, the impact of PCaAl-LDHs on the moisture content of the treated materials was negligible, and its influence on the thickness of water absorption-induced swelling was significantly lower than that of other inorganic flame retardants based on water. Thus, we posited that the PCaAl-LDHs synthesized in this investigation possessed the potential to reduce the drawbacks of water-based inorganic flame retardants’ susceptibility to hygroscopicity. The slight impact of the flame-retardant treatment on the water absorption and expansion rate of the bamboo scrimber could bring the following benefits. Firstly, it significantly contributed to maintaining the dimensional stability of the bamboo scrimber, reducing the deformation and cracking caused by water absorption. Moreover, it could reduce the damage caused by humid environments to the bamboo scrimber and prolong its service life. Lastly, it played a crucial role in preserving the fire-retardant capabilities of the flame retardant, consequently reducing the potential risks associated with fire hazards. Overall, the little impact on the water absorption and expansion of treated materials experienced advantages in dimensional stability, durability, and fire safety.

### 2.4. Flame Retardant Property of FRBS

**Figure 5:**
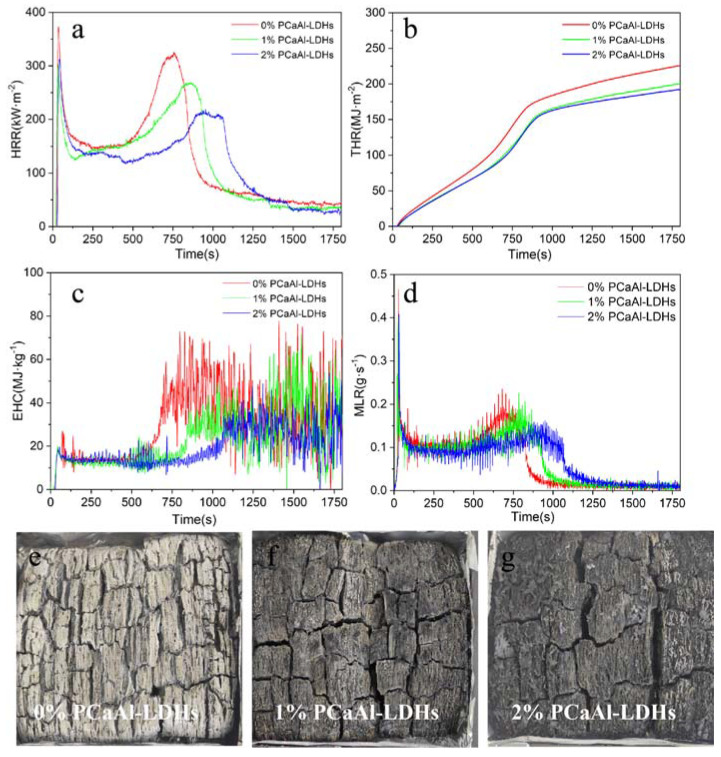
The cone calorimetric test results and carbon residues digital photos of FRBS. (**a**) HRR, (**b**) THR, (**c**) EHC, (**d**) MIL, (**e**) carbon residues of raw bamboo, (**f**) carbon residues of flame retardant concentration 1%, (**g**) carbon residues of flame retardant concentration 2%.

To evaluate the flame retardancy of FRBS, conical calorimetry tests were performed on the bamboo scrimber treated with different concentrations of flame retardants and raw bamboo scrimber. The heat release rate (HRR) refers to the heat released per unit of time during the combustion of materials, which is one of the most important parameters for determining the fire risk of materials [19]. As shown in Figure 5a, the peaks of the exothermic heat values of raw bamboo scrimber, including 1% flame retardant FRBS and 2% flame retardant FRBS, were 325, 267, and 213 kw·m^2^, respectively. Compared with the untreated bamboo scrimber, the HRR of 1% and 2% treated bamboo scrimber decreased by 17.58% and 34.46%, respectively. These results showed that PCaAl-LDHs effectively inhibited the combustion of bamboo scrimber and effectively improved the fire safety of the bamboo scrimber.

The total heat release (THR) is a sum parameter describing the total heat release per unit area of a sample from the beginning to the end of combustion [20]. Combining THR with HRR can more comprehensively evaluate the combustion characteristics of the material [21]. As can be seen from Figure 5b, during the whole combustion process, the THR of FRBS increased with the extension of time. Compared with the original, the THR of FRBS under different flame-retardant concentrations was reduced by 11.89% and 15.86%, respectively, which was consistent with the variation in the HRR curve. This further indicated that the addition of PCaAl-LDHs could improve the flame-retardant property of bamboo scrimber and reduce the risk of building fire.

Figure 5c shows the effective heat combustion (EHC) of the bamboo scrimber treated with different concentrations of flame retardants [22,23]. The EHC of FRBS treated with 1% and 2% concentrations were lower than that of the untreated bamboo scrimber. The mass loss rate (MLR) represented the rate of the loss mass during combustion experiment [24]. Figure 5d shows the peak value of the mass loss rate of FRBS treated with a 1% and 2% flame retardant concentration, which were delayed by 120 s and 236 s, respectively. The study found that the bamboo scrimber produced through this method could significantly slow down the combustion, decrease the rate of pyrolysis reaction in the bamboo, and allow for sufficient time to safely evacuate people and materials during a fire [25,26].

As can be seen from Figure 5e–g, the residual material after the combustion of untreated bamboo scrimber was mainly gray powder ash, and the residual carbon of FRBS was black and gray. This indicated that the addition of PCaAl-LDHs could promote the formation of a stable, dense, and regular carbon layer structure, which could effectively inhibit heat transfer in the combustion process [7,27]. In conclusion, this method could greatly improve the fire resistance of the bamboo scrimber and reduce the fire hazard.

### 2.5. Smoke Suppression Property of FRBS

Smoke production increased the risk of asphyxiation, which was often more deadly than the heat from the fire [9]. Due to the protection of the carbon layer, combustible gas and smoke-forming materials could rapidly decrease in the gas phase during combustion [28,29]. The total smoke released the curve that consisted of two stages: the initial flame combustion process and the later smoldering process [30,31].

**Figure 6:**
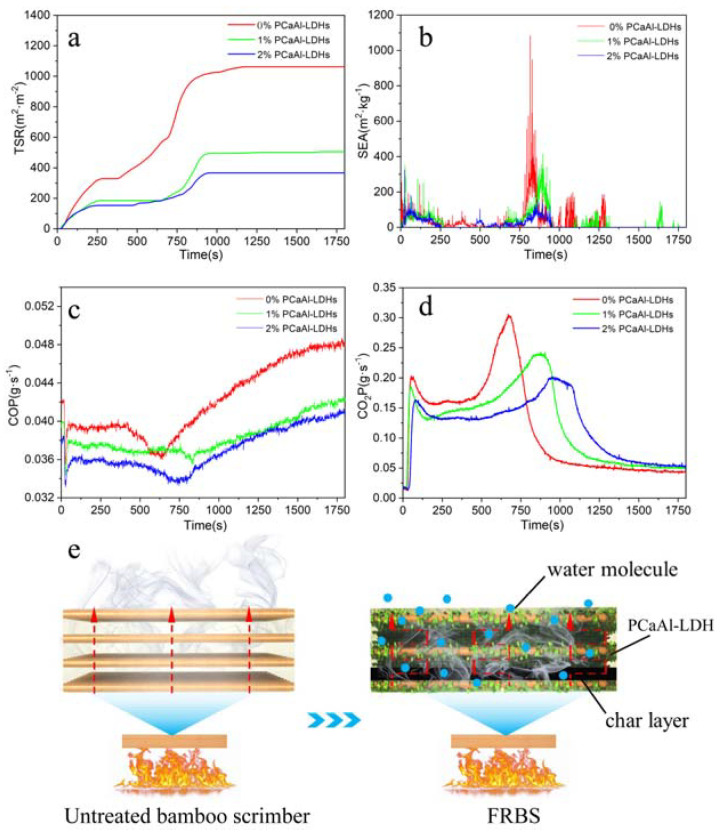
The smoke suppression properties of FRBS. (**a**) TSR, (**b**) SEA, (**c**) COP, (**d**) CO_2_P, (**e**) Smoke suppression mechanism of FRBS.

Figure 6a clearly shows that the untreated bamboo scrimber had a significantly higher TSR than FRBS throughout the entire combustion process. At the initial stage of the combustion test, the TSR of all samples increased with the extension of time, and then the TSR curve became gentle. However, in the later smoldering process, the TSR of all samples began to increase with the extension of time. The TSR of 1% and 2% FRBS decreased by 52.74% and 65.97% compared with that of the untreated bamboo scrimber. The specific extinction area (SEA) represented the ability to volatize the smoke produced by each unit mass of fuel, which could measure the shading property of smoke [32]. The larger the value was, the larger the smoke produced by the volatile materials [33,34]. As depicted in Figure 6b, the SEA value of the original material exhibited a consistently higher trend than that of the FRBS treated with a 1% and 2% concentration for the majority of the observed duration. The peak value of the SEA curve of the original in the second flame-burning stage was higher than FRBS. In addition, the smoke emission at this time was the main cause of the smoke hazards in the fire. The peak value of the original was 1040 m^2^·kg^−1^, and the peak value of FRBS with 1% and 2% concentration was 426 m^2^·kg^−1^ and 146 m^2^·kg^−1^, respectively. Compared with the original, the peak value of FRBS decreased by 59.04% and 85.96%, corresponding to the concentration of 1% and 2%, respectively. These results indicate that the bamboo scrimber treated with PCaAl-LDHs had a significant smoke suppression effect.

Controlling smoke generation is of great significance for improving fire safety [33]. After conducting the cone calorimeter test, it was evident from Figure 6c,d that both COP and CO_2_P of FRBS were lower than those of the original. Compared to the raw, the peak arrival time of FRBS with a 1% and 2% concentration was delayed by 3.3 min and 6.4 min, respectively. Figure 6e roughly describes the flame retardancy and smoke suppression mechanism of FRBS. FRBS demonstrated a good flame retardant and smoke suppression performance, which was mainly due to the following three reasons [35,36]. Firstly, the unique two-dimensional laminar structure of PCaAl-LDHs prolonged the diffusion path of flammable gas in the shape of “Z”. Secondly, the oxidation of PCaAl-LDHs to transition metal oxides accelerated the formation of the carbon layers, thus effectively inhibiting the diffusion of flue gas, while isolating external heat and the oxygen slowed the combustion rate [37,38]. Finally, PCaAl-LDHs could release interlayer water molecules at a high temperature, which could reduce the temperature and dilute the concentration of combustible gas [39,40], which was significantly slow down the combustion rate and effectively inhibited gas release.

## 3. Materials and Methods

### 3.1. Materials

Aluminum nitrate nine-hydrate (Al(NO_3_)_3_‧9 H_2_O), Calcium nitrate tetrahydrate (Ca(NO_3_)_2_‧4 H_2_O), Sodium phosphate (Na_3_PO_4_), and Sodium hydroxide (granular) (NaOH) were all purchased from the Sinopharm Group Chemical Reagent Co., Ltd. (China), and the specifications were all analytically pure. Phenolic resin adhesive (solid content 52.3%, pH = 10.5) was purchased from Shandong Tianzhao Chemical Industry Group Co., Ltd. (Shandong, China). The uncharred bamboo bundle was purchased from Anji Yongyu Bamboo Co., Ltd. (Zhejiang, China).

### 3.2. Preparation of PCaAl-LDHs Flame-Retardant Bamboo Scrimber (FRBS)

In this section, Na_3_PO_4_ was utilized as the source of the interlayer anions required for the intercalation process. By employing the coprecipitation method under specific process conditions of the crystallization temperature of 120 °C and a crystallization time of 6 h, the flame retardant PCaAl-LDHs with an excellent flame retardant performance was successfully synthesized. The specific synthesis process of the PCaAl-LDHs could be referred to in our previous work [41]. The rough surface structure of bamboo bundles endowed them with exceptional adsorption properties. Taking advantage of this characteristic, a simple room temperature and atmospheric pressure impregnation method was employed to attach the PCaAl-LDH onto the surface of the bamboo bundles.

**Figure 7:**
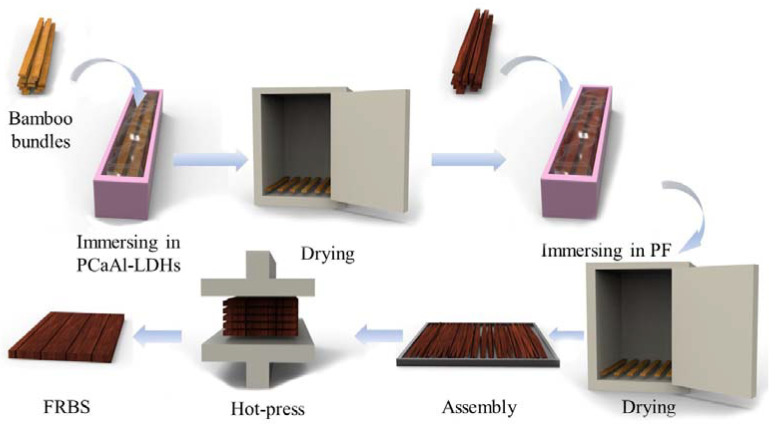
The manufacturing and preparation process of FRBS.

The preparation process of the FRBS is shown in Figure 7. Firstly, the PCaAl-LDH flame retardant prepared in the previous step was dispersed in an aqueous solution at room temperature through ultrasonic dispersion. The effects of the flame-retardant agent concentration and immersion time on the drug loading ratio of bamboo bundles were investigated. Specifically, the gradient of flame retardant concentrations was set at 1%, 2%, 3%, and 4%, while the gradient of the immersion time was set at 1 h, 2 h, 3 h, and 4 h. After achieving the desired process conditions for the bamboo bundles’ impregnation treatment, the bamboo bundles were removed and dried in an oven until completely dry, and then set aside for further use. Second, the phenolic resin adhesive with a solid content of 52.3% was diluted with water to a solid content of 26%. The bamboo bundles, after flame retardant treatment, were soaked in a phenolic resin adhesive solution for 8 min. After the leaching glue was removed, the bamboo bundles were dried in the oven at 65 °C until the moisture content was 11%~14%. Then, the flame retardant bamboo bundles coated with phenolic resin were placed in a customized mold (length × width × height: 48 cm × 41 cm × 1.5 cm), and the slab was laid by hand along the pattern. Finally, the slab was placed in a hot press and was pressed to obtain flame retardant bamboo scrimber under the process conditions of a hot pressing pressure 4.0 MPa, 140 °C, 1.5 min·mm^−1^, and the target density of the bamboo scrimber was 1 g·cm^−3^. The non-flame retardant bamboo scrimbers were pressed by the same process for comparative study.

### 3.3. Scanning Electron Microscopy

The microstructures of bamboo bundles before and after dipping were observed with a SU8010 cold field emission scanning electron microscope (SEM) manufactured by Hitachi.

### 3.4. Energy Dispersive X-ray Spectrometry

Based on SEM, the element composition of the bamboo bundles after flame retardant treatment was detected and analyzed under the acceleration voltage of 10 kV.

### 3.5. Physical and Mechanical Properties Tests

It is crucial to take into account the influence of the flame retardant on the physical and mechanical properties of the bamboo scrimber. This consideration is of paramount importance in order to ensure the overall performance and functionality of the materials in various applications. According to the Chinese national standard “Reconstituted Bamboo Floor” (GB/T 30364–2013), the density, water absorption expansion rate, horizontal shear strength, and other physical and mechanical properties of FRBS and non–FRBS were investigated. The effects of adding flame retardants on the physical and mechanical properties of the bamboo scrimber were compared and analyzed.

### 3.6. Cone Calorimetry Test

FRBS and non–FRBS size specifications for 100 mm × 100 mm. The cone calorimeter (CONE) was produced by the German Nestane Instrument Manufacturing Co., Ltd (STA409C). The thermal radiation power was 50 KW·m^−2^, while the airflow was 24 L·s^−1^.

## 4. Conclusions

This work successfully fabricated FRBS by compositing PCaAl-LDHs with bamboo bundles, which was validated by SEM, EDS, and CONE measurements. The results indicated that PCaAl-LDHs flame retardants were uniformly distributed on the bamboo bundles. In addition, when the flame−retardant concentration was 2%, the HRR and THR of the as prepared FRBS were, respectively, reduced by 34.46% and 15.86% in comparison with the untreated bamboo scrimber. Furthermore, FRBS not only had excellent flame retardant properties but also significant smoke suppression properties. Cone calorimetry showed that the total smoke emission (TSR) and specific extinction area (SEA) of FRBS were reduced by 65.97% and 85.96% when the concentration of the flame retardant was 2%, which greatly improved the fire safety of the bamboo scrimber. The mechanism analysis showed that the two—dimensional lamellae structure of PCaAl-LDHs extended the flue gas diffusion path, the catalytic carbonization of metal oxides promoted the formation of a dense carbon layer, effectively isolating external oxygen and heat, and the cooling and dilution effects of water molecules released between the layers were key reasons for the improvement of the flame retardant and smoke suppression performance of the bamboo scrimber. In conclusion, this groundbreaking method not only enhances the fire safety of bamboo scrimber but also opens up a world of new possibilities for its application.

## Figures and Tables

**Table 1:** Physical properties of bamboo scrimber treated with different flame retardants [18].

Sample Number	Drug Loading Rate/%	Water Content/%	Swelling Rate of Absorbent Thickness/%
1	3.98	39.5	300
2	4.81	0.9	103.95

## Data Availability

Not applicable.
